# Sparse Phenotyping and Haplotype-Based Models for Genomic Prediction in Rice

**DOI:** 10.1186/s12284-023-00643-2

**Published:** 2023-06-07

**Authors:** Sang He, Shanshan Liang, Lijun Meng, Liyong Cao, Guoyou Ye

**Affiliations:** 1 Shenzhen Branch, Guangdong Laboratory of Lingnan Modern Agriculture, Genome Analysis Laboratory of the Ministry of Agriculture and Rural Affairs, Agricultural Genomics Institute at Shenzhen, Chinese Academy of Agricultural Sciences, Shenzhen, 518124 China; 2 CAAS-IRRI Joint Laboratory for Genomics-Assisted Germplasm Enhancement, Agricultural Genomics Institute at Shenzhen, Chinese Academy of Agricultural Sciences, Shenzhen, 518124 China; 3 Tianjin Key Laboratory of Animal and Plant Resistance, College of Life Sciences, Tianjin Normal University, Tianjin, 300387 China; 4 Kunpeng Institute of Modern Agriculture at Foshan, Foshan, 528200 China; 5 Key Laboratory for Zhejiang Super Rice Research, China National Rice Research Institute, Hangzhou, 310006 China; 6 Rice Breeding Innovations Platform, International Rice Research Institute, Metro Manila, Philippines

**Keywords:** Sparse phenotyping, Training set, Linkage disequilibrium, Haplotype-based model, Genomic prediction, Rice

## Abstract

The multi-environment genomic selection enables plant breeders to select varieties resilient to diverse environments or particularly adapted to specific environments, which holds a great potential to be used in rice breeding. To realize the multi-environment genomic selection, a robust training set with multi-environment phenotypic data is of necessity. Considering the huge potential of genomic prediction enhanced sparse phenotyping on the cost saving of multi-environment trials (MET), the establishment of a multi-environment training set could also benefit from it. Optimizing the genomic prediction methods is also crucial to enhance the multi-environment genomic selection. Using haplotype-based genomic prediction models is able to capture local epistatic effects which could be conserved and accumulated across generations much like additive effects thereby benefitting breeding. However, previous studies often used fixed length haplotypes composed by a few adjacent molecular markers disregarding the linkage disequilibrium (LD) which is of essential role in determining the haplotype length. In our study, based on three rice populations with different sizes and compositions, we investigated the usefulness and effectiveness of multi-environment training sets with varying phenotyping intensities and different haplotype-based genomic prediction models based on LD-derived haplotype blocks for two agronomic traits, i.e., days to heading (DTH) and plant height (PH). Results showed that phenotyping merely 30% records in multi-environment training set is able to provide a comparable prediction accuracy to high phenotyping intensities; the local epistatic effects are much likely existent in DTH; dividing the LD-derived haplotype blocks into small segments with two or three single nucleotide polymorphisms (SNPs) helps to maintain the predictive ability of haplotype-based models in large populations; modelling the covariances between environments improves genomic prediction accuracy. Our study provides means to improve the efficiency of multi-environment genomic selection in rice.

**Supplementary Information:**

The online version contains supplementary material available at 10.1186/s12284-023-00643-2.

## Background

Rice is a staple crop feeding more than half of the human population (Londo et al. 2006; Muthayya et al. 2014). Sustaining and improving the selection gain of yield as well as other pivotal traits critical to human health to meet the huge demand of people in the future is an arduous task for rice breeders. Conventional plant breeding based solely on phenotypic selection is not capable enough to meet the request. Contrastingly, genomics-assisted breeding has shown its great potential in improving the efficiency of plant breeding (Crossa et al. 2017; Endelman et al. 2014; Xu et al. 2021). In the early stages of breeding programs, using genomic selection, a representative genomics-assisted breeding approach, is able to improve the selection accuracy relative to conventional phenotypic selection (Endelman et al. 2014; He et al. 2016). In the middle stages, multi-environment trials are commonly deployed and genomic selection loses its superiority to phenotypic selection on selection accuracy (Atanda et al. 2022). Therefore, a straightforward use of genomic estimated breeding values (GEBVs) from genomic selection to identify elite candidates is no longer advantaged. Despite this, plant breeding can also be assisted by genomics through a genomic prediction enhanced sparse phenotyping method proposed by Jarquín et al. (2020). Specifically, a complete phenotypic evaluation of total selection candidates in all environments in MET is cost-intensive and not necessitated. Instead, with the help of genomic prediction, applying a sparse phenotyping only evaluating a subset of candidates in some environments to reduce the overall budget or expanding the total evaluation capacity with a fixed cost is more cost-efficient and worth to be applied (He et al. 2021; Jarquín et al. 2020). The vacant phenotypes can be reliably predicted by the observed records using genomic prediction based on the relatedness between the selection candidates and the correlation between trials or environments. Considering the pivotal role of training set in genomic prediction, the sparse phenotyping can also be used in establishing a multi-environment training set. The multi-environment training set enables the prediction of selection candidates’ performances in a specific environment, which is of value and interest to plant breeders to breed varieties resilient to multiple environments or particularly adapted to a specific environment. The sparse phenotyping approach could make the multi-environment training set establishment more cost-efficient. To our knowledge, no similar investigation has been reported in rice.

The local epistatic effect existing between adjacent molecular markers, e.g., SNPs, has a high chance to be conserved and accumulated over breeding generations, which is much like additive effect and worth to be accommodated in genomic prediction (Akdemir and Jannink 2015; Jiang et al. 2018). The haplotype-based genomic prediction approach has been theoretically proven to be able to explicitly and efficiently capture the local epistasis (Jiang et al. 2018). Previous studies mostly straightforwardly set haplotype blocks with a fixed length consisting of a few adjacent markers (He et al. 2017, 2019; Jiang et al. 2018). However, this presumption is too arbitrary as in fact the length of haplotype block is varying depending on the LD among the adjacent SNPs. The haplotype blocks identified in a diverse population with genetically distant individuals are mostly short while in a biparental population the blocks could be long. Therefore, it is more reasonable to condition the haplotype blocks on LD to realistically capture the inheritable local epistatic effects. On the other hand, relying on LD could also produce long haplotype blocks with a large number of haplotype alleles. If the population is small, a great proportion of haplotypes would have low frequencies. These rare haplotypes would be excluded from analyses by the quality control of genomic data, resulting in a loss of genetic information. To cope with it, the haplotype blocks defined by LD could be further divided into small fragments composed by a few SNPs, e.g., two or three SNPs. By this way, both the resilience in constructing haplotype blocks based on LD and the risk of losing genetic information are considered, thereupon the local epistatic effects could be effectively modelled in genomic prediction.

In our study, we based on three rice inbred line populations and two agronomic traits DTH and PH to investigate 1) the potential of using genomic prediction enhanced sparse phenotyping to establish a cost-efficient multi-environment training set, and 2) whether using the haplotype-based genomic prediction models based on LD-derived haplotype blocks could improve the prediction accuracy relative to the marker-based approaches in rice.

## Materials and Methods

### Rice Populations and Data Processing

The first population is from Spindel et al. (2015) including 344 elite breeding lines fingerprinted by 108,024 SNPs. These lines were phenotyped for DTH and PH in dry season (DS) and wet season (WS) across four years using a randomized complete block design with three replicates. The combination of season and year was termed as environment. The SNP markers were discovered and called from the TASSEL3.0 GBS pipeline (Glaubitz et al. 2014). Heterozygous marker scores were all set to missing. SNPs with minor allele frequency less than 0.05 or missing rate greater than 0.1 were removed by the quality control of raw genotypic data. Finally, 44,116 SNPs were available for the 344 genotyped lines.

The second population comes from Liang et al. (2015) incorporating 254 lines genotyped by 49,988 SNPs. These lines were phenotypically tested for DTH and PH in two DS and two WS with different nitrogen fertilizer application rates. The combination of season and nitrogen fertilizer application rate was regarded as the environment. Eventually, phenotypic data of four environments was accessible for the 254 lines. The genotypic data was first filtered by assigning all heterozygous SNP scores to missing values and then quality controlled by removing SNPs with minor allele frequency less than 0.05 or missing rate greater than 0.1. Ultimately, 1193 SNPs were available for the 254 lines.

The third population is provided by Meng et al. (2016) and Qu et al. (2020) comprising 1048 recombinant inbred lines (RILs) originating from eight genetically distant *Indica* lines. These RILs were phenotypically evaluated for DTH and PH in DS and WS of year 2014 in International Rice Research Institute (IRRI) and two locations in China, namely Jiangxi (JX) and Shenzhen (SZ), in year 2016. The combination of season/location and year was regarded as the environment, thereupon phenotypic data of four environments was available for the 1048 RILs. These RILs were fingerprinted by a customized rice 55 K SNP array (Qu et al. 2020). After assigning all heterozygous SNP scores to missing data and the quality control of removing SNPs with minor allele frequency less than 0.05 or missing rate greater than 0.1, 33,518 SNPs were available for the 1048 RILs.

For the first and second populations, a two-stage phenotypic analysis was implemented to derive the repeatability of each environment and best linear unbiased estimates (BLUEs) of genetic effects of all lines. Specifically, in the first stage, the spatial adjustment of field trial data was conducted in each environment by fitting the model: ${\varvec{y}} = 1_{i} \mu + {\varvec{X}}_{{\varvec{w}}} {\varvec{w}} + {\varvec{Z}}_{{\varvec{l}}} {\varvec{l}} + {\varvec{\epsilon}}$, where ${\varvec{y}}$ is a i-dimensional vector of phenotypic records across environments, i is the number of phenotypic records across environments, $\mu$ is the intercept, $1_{i}$ is an i-dimensional vector of ones, ${\varvec{w}}$ is the vector containing experimental design effects, ${\varvec{l}}$ is the vector of environment-specific genetic effects of lines, ${\varvec{X}}_{{\varvec{w}}}$ and ${\varvec{Z}}_{{\varvec{l}}}$ are design matrices for ${\varvec{w}}$ and ${\varvec{l}}$, ${\varvec{\epsilon}}$ is the random residual. The genetic effect of lines was respectively treated as a fixed and a random effect to derive the environment-specific BLUEs of genetic effects of lines and repeatability of each environment. The experimental design effects such as replicate, column, and row were all regarded as random effects. In the second stage, the environment-specific BLUEs were combined and a linear model including environment main effect and genetic effect was fitted using formula: $\hat{\varvec{y}} = 1_{n} \mu + {\varvec{Z}}_{{\varvec{v}}} {\varvec{v}} + {\varvec{Z}}_{{\varvec{g}}} {\varvec{g}} + {\varvec{e}}$, where $\hat{\varvec{y}}$ is n-dimensional vector of BLUEs of genetic effects of lines across environments, $1_{n}$ is a n-dimensional vector of ones, $\mu$ is the intercept, ${\varvec{v}}$ is the vector of environment main effects regarded as a random effect, ${\varvec{g}}$ is the vector of genetic effects of lines regarded as a random effect. ${\varvec{Z}}_{{\varvec{v}}}$ and ${\varvec{Z}}_{{\varvec{g}}}$ are the design matrices for ${\varvec{v}}$ and ${\varvec{g}}$, ${\varvec{e}}$ is the random residual. All random effects in the first and second stages were assumed to follow identical and independent normal distributions that could be uniformly expressed as $\phi \sim N\left( {0, {\varvec{I}}\sigma_{\phi }^{2} } \right)$ where $\phi$ is the random effect concerned, ${\varvec{I}}$ is an identity matrix, and $\sigma_{\phi }^{2}$ is corresponding variance component. The repeatability in each environment and heritability of trait are both estimated using formula: $1 - \frac{{\overline{c}}}{{2\sigma^{2} }}$, where $\overline{c}$ is the mean variance of a difference between two best linear unbiased predictions (BLUP) of genetic effects of lines, $\sigma^{2}$ is the variance component of genetic effect (Cullis et al. 2006). It is a generalized measure of heritability which relates to the response to selection even when field trial data is unbalanced as compared to the standard measure based on the variance explanation (Cullis et al. 2006; Falconer and Mackay 1996). The third population only has the spatially adjusted phenotypic values of RILs in each environment thus the second stage analysis was merely implemented. The phenotypic analysis models were performed in R (R Core Team 2016) using R package sommer (Covarrubias-Pazaran 2016).

### Haplotype Block and Haplotypic Data

To infer the haplotypes, the genotypic data after quality control was specifically imputed and phased using SHAPEIT software (Delaneau et al. 2012). The haplotype blocks were detected from the phased genotypic data based on LD using PLINK software (Chang et al. 2015) with the flag “–blocks” with the default settings including 1) the LD was assessed between SNPs within a 200 kilobase window; 2) block was formed if 95% of informative SNP pairs were in strong LD; the strong LD being defined as the 90% confidence interval for D-prime, considering the pairwise LD was between 0.7 and 0.98 (Chang et al. 2015; Gabriel et al. 2002). The identified haplotype blocks were further divided into small fragments with a fixed length of two and three SNPs respectively. The genotypic scores of the haplotypic data with complete haplotypic block were the number of copies of each haplotype in the haplotype blocks. The genotypic scores of small haplotype fragments with a fixed length were the number of copies of haplotype in the fragments.

### Multi-Environment Genomic Prediction Approaches

Three multi-environment genomic prediction models were used in our study. The genetic effect in all models was respectively described by marker genotypes (marker-based model) and haplotypes (haplotype-based model). Following variables were identically defined in all models: $\hat{\varvec{y}}$ is the n-dimensional vector containing BLUEs of genetic effects of lines in each environment, n is the number of BLUEs across environments, $1_{n}$ is a n-dimensional vector of ones, $\mu$ is the intercept, ${\varvec{v}}$ is the vector of environment main effects, ${\varvec{g}}$ is the vector of additive genetic effects of lines for marker-based model or additive plus local epistatic genetic effects of lines for haplotype-based model. ${\varvec{Z}}_{{\varvec{v}}}$ and ${\varvec{Z}}_{{\varvec{g}}}$ are the design matrices for ${\varvec{v}}$ and ${\varvec{g}}$, ${\varvec{e}}$ is the random residual. ${\varvec{v}}$, ${\varvec{g}}$, and ${\varvec{e}}$ were all assumed as random effects following ${\varvec{v}}\sim N\left( {0, {\varvec{I}}\sigma_{v}^{2} } \right)$, ${\varvec{g}}\sim N\left( {0,{\varvec{K}}\sigma_{g}^{2} } \right)$ and ${\varvec{e}}\sim N\left( {0, {\varvec{I}}\sigma_{e}^{2} } \right)$ where ${\varvec{I}}$ is an identity matrix, $\sigma_{v}^{2}$, $\sigma_{e}^{2}$ and $\sigma_{g}^{2}$ are the corresponding variance components. The genomic relationship matrix $${\varvec{K}} = \left\{ {\begin{array}{*{20}l} {G\;marker - based\;model} \hfill \\ {H\;haplotype - based\;model} \hfill \\ \end{array} } \right.$$ was estimated following Jarquín et al. (2014). In marker-based models, the relationship matrix ${\varvec{G}}{ }$ was established solely using the SNP marker scores in which the scattered missing scores per SNP were naively imputed using the mean value of entries fingerprinted. For haplotype-based models, the relationship matrix ${\varvec{H}}$ was a combined matrix of relationship matrix based on marker scores of SNPs not included in any haplotype blocks and haplotypic relationship matrix derived from the haplotypes. The entries in ${\varvec{K}}$ are given by $K_{{ii^{\prime}}} = \sum\nolimits_{m = 1}^{p} {\frac{{\left( {x_{im} - 2\delta_{m} } \right)\left( {x_{{i^{\prime}m}} - 2\delta_{m} } \right)}}{{2\delta_{m} \left( {1 - \delta_{m} } \right)}}/p}$ where $x_{im}$ is the number of copies of alternative allele of m^th^ SNP in i^th^ line when marker scores were used, i.e., SNPs in marker-based models and those not included in any haplotype blocks in haplotype-based models, or m^th^ haplotype carried by i^th^ line when haplotypes were used, i.e., haplotypes in haplotype-based models, $\delta_{m}$ is the frequency of alternative allele of m^th^ SNP or m^th^ haplotype, *p* is the total number of SNPs in marker-based models or the number of SNPs not included in any haplotype blocks plus the amount of haplotypes in haplotype-based models.

The first model considers no genotype-by-environment interaction formulated as

VG $$\hat{\varvec{y}} = 1_{n} \mu + {\varvec{Z}}_{{\varvec{v}}} {\varvec{v}} + {\varvec{Z}}_{{\varvec{g}}} {\varvec{g}} + {\varvec{e}}$$

The second model explicitly portrays genotype-by-environment interaction as

VGR $$\hat{\varvec{y}} = 1_{n} \mu + {\varvec{Z}}_{{\varvec{v}}} {\varvec{v}} + {\varvec{Z}}_{{\varvec{g}}} {\varvec{g}} + {\varvec{r}} + {\varvec{e}}$$

where ${\varvec{r}}$ is a n-dimensional vector of genotype-by-environment interaction effects following ${\varvec{r}}\sim N\left( {0,{\varvec{Z}}_{{\varvec{v}}} {\varvec{Z}}_{{\varvec{v}}}^{\varvec{^{\prime}}} \circ {\varvec{Z}}_{{\varvec{g}}} {\varvec{KZ}}_{{\varvec{g}}}^{\varvec{^{\prime}}} \sigma_{r}^{2} } \right)$ where $\circ$ denotes the Hadamard product of matrices and $\sigma_{r}^{2}$ is the variance component of genotype-by-environment interaction effect.

The third model is the factorial analytic (FA) model (Burgueño et al. 2012; Smith et al. 2001) able to accommodate the covariances between environments, formulated as

FA $$\hat{\varvec{y}} = {\varvec{\mu}} + {\varvec{u}} + {\varvec{\varepsilon}}$$

where ${\varvec{\mu}} = \left( {{\varvec{\mu}}_{1}^{{\mathbf{\prime }}} , \ldots , {\varvec{\mu}}_{{\varvec{j}}}^{{\mathbf{\prime }}} , \ldots ,{\varvec{\mu}}_{{\varvec{l}}}^{{\mathbf{\prime }}} } \right)^{{\mathbf{\prime }}}$, ${\varvec{u}} = \left( {{\varvec{g}}_{1}^{{\mathbf{\prime }}} , \ldots , {\varvec{g}}_{{\varvec{j}}}^{{\mathbf{\prime }}} , \ldots ,{\varvec{g}}_{{\varvec{l}}}^{{\mathbf{\prime }}} } \right)^{{\mathbf{\prime }}}$, ${\varvec{\varepsilon}} = \left( {{\varvec{e}}_{1}^{{\mathbf{\prime }}} , \ldots ,{\varvec{e}}_{{\varvec{j}}}^{{\mathbf{\prime }}} , \ldots ,{\varvec{e}}_{{\varvec{l}}}^{{\mathbf{\prime }}} } \right)^{{\mathbf{\prime }}}$, ${\varvec{\mu}}_{{\varvec{j}}}^{{\mathbf{\prime }}}$, ${\varvec{g}}_{{\varvec{j}}}^{{\mathbf{\prime }}}$ and ${\varvec{e}}_{{\varvec{j}}}^{\varvec{^{\prime}}}$ are the vectors of intercept, genetic effects and residuals in j^th^ environment. We assumed ${\varvec{u}}\sim N\left( {0,{\varvec{\varPsi}}_{{\varvec{u}}} \otimes {\varvec{K}}} \right)$,$\varvec{ \varepsilon }\sim N\left( {0,{\varvec{\varPsi}}_{{\varvec{\varepsilon}}} \otimes {\varvec{I}}} \right)$, where $${\varvec{\varPsi}}_{{\varvec{u}}} = \left( {\begin{array}{*{20}c} {\sigma_{{g_{1} }}^{2} } & \cdots & {cov_{{g_{1} g_{j} }} } & \cdots & {cov_{{g_{1} g_{l} }} } \\ \vdots & \ddots & \vdots & \ddots & \vdots \\ {cov_{{g_{j} g_{1} }} } & \cdots & {\sigma_{{g_{j} }}^{2} } & \cdots & {cov_{{g_{j} g_{l} }} } \\ \vdots & \ddots & \vdots & \ddots & \vdots \\ {cov_{{g_{l} g_{1} }} } & \cdots & {cov_{{g_{l} g_{j} }} } & \cdots & {\sigma_{{g_{l} }}^{2} } \\ \end{array} } \right)$$ is the variance–covariance matrix of genetic effect of lines across environments, $\otimes$ denotes the Kronecker product of matrices, $\sigma_{{g_{j} }}^{2}$ is the genetic variance of j^th^ environment, $cov_{{g_{j} g_{l} }}$ denotes the genetic covariance between environment j and l. In FA model, the variance–covariance matrix is ${\varvec{\varPsi}}_{{\varvec{u}}} = \left( {\varvec{\Lambda \Lambda }^{{\mathbf{\prime }}} +{\varvec{\varPi}}} \right) = {\text{FA}}\left( {\text{t}} \right)$ in which t is the number of latent factors, ${\varvec{\varLambda}}$ is a l × t-dimensional matrix containing environment loadings, ${\varvec{\varPi}}$ is a l × l diagonal matrix (Burgueño et al. 2012), $${\varvec{\varPsi}}_{{\varvec{\varepsilon}}} = \left( {\begin{array}{*{20}c} {\sigma_{{e_{1} }}^{2} } & \cdots & 0 & \cdots & 0 \\ \vdots & \ddots & \vdots & \ddots & \vdots \\ 0 & \cdots & {\sigma_{{e_{j} }}^{2} } & \cdots & 0 \\ \vdots & \ddots & \vdots & \ddots & \vdots \\ 0 & \cdots & 0 & \cdots & {\sigma_{{e_{l} }}^{2} } \\ \end{array} } \right)$$ where $\sigma_{{e_{j} }}^{2}$ is the residual variance of j^th^ environment. In our study, we specified t = 1 (one latent factor) in all the three populations.

All the models were implemented in R (R Core Team 2016). VG and VGR models were realized using R package BGLR (Pérez and de los Campos 2014). FA was implemented in R package MTM (de los Campos and Grüneberg 2016). Bayesian algorithm was used to estimate the model components. The number of iterations in all models were set to 10,000 and first 4000 iterations were discarded as burn-in.

### Cross-Validation and Sparse Phenotyping Scheme

**Fig. 1:**
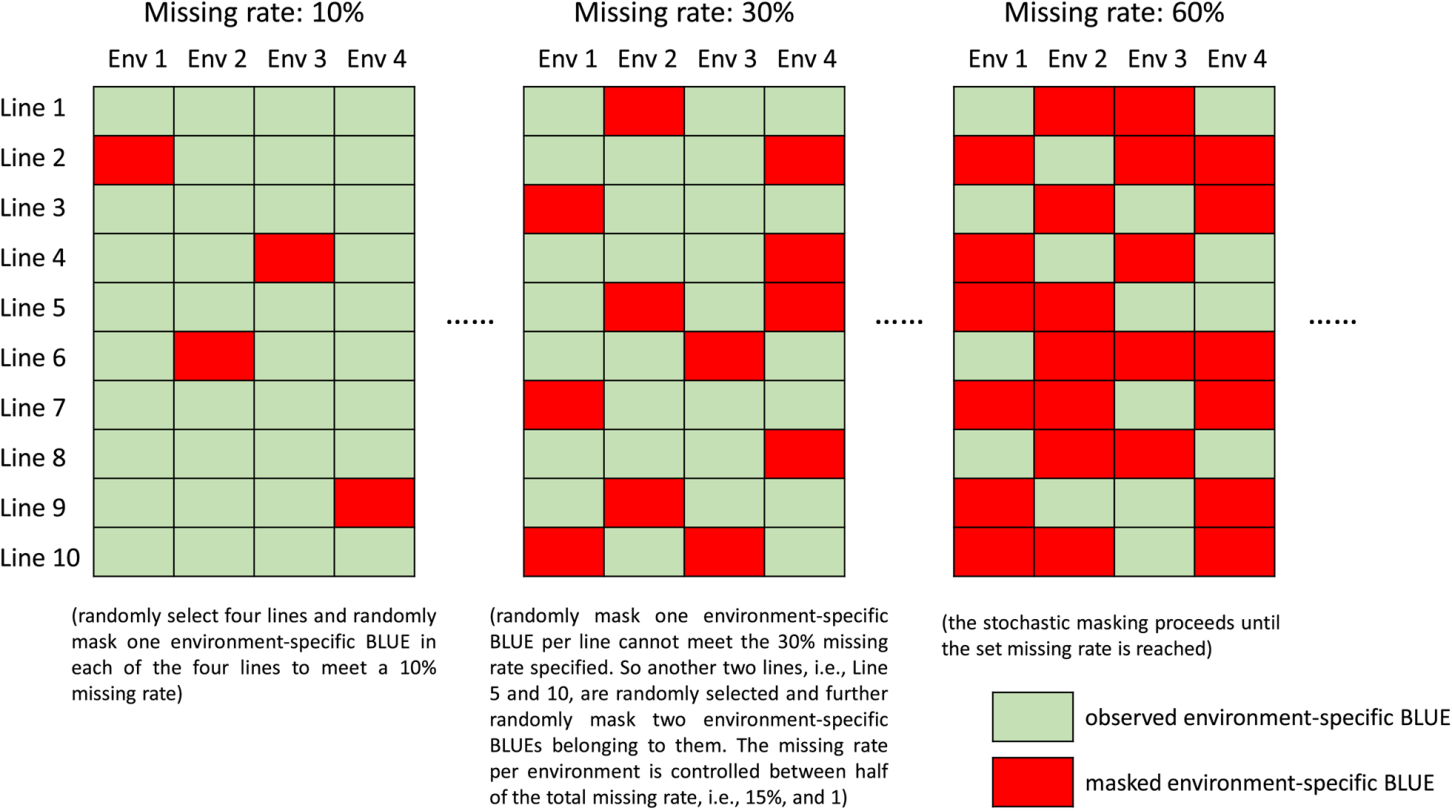
The procedure of generating sparse phenotypes in the multi-environment training set

We used the first cross-validation strategy (CV1) with 5 folds in Burgueño et al. (2012) to assess the genomic prediction accuracy. It mimicked the situation of predicting newly developed lines that have never undergone field test in breeding. All genotyped lines were randomly divided in two 5 equal-size folds. Four folds were combined as the training set and the remaining fold was the test set. In each division of training and test sets, the procedure of generating sparse phenotypes in the training set was: the lines in the training set were shuffled and one environment-specific BLUE per line was randomly selected and masked attempting to reach the missing rate specified. If masking one BLUE of all lines was insufficient to reach the missing rate, another BLUE of some lines were randomly picked and marked until the missing rate was reached. To balance the volume of available phenotypic information in each environment, the stochastic masking process of the environment-specific BLUEs of all environments was looped until the missing rate per environment was larger than half of total missing rate and smaller than 1. The detailed procedure of generating the sparse phenotypes was illustrated in Fig. 1. The missing rate ranged from 10 to 90%. The process of stochastically masking phenotypes under each missing rate was repeated 10 times. For comparison, the training sets with complete environment-specific BLUEs were also used to train the genomic prediction models.

The 5-fold cross-validation was repeated 10 times, yielding 50 divisions of training and test sets. Finally, there were totally five hundred times (5 × 10 × 10) calibrations and predictions for each training set missing rate (10–90%). Considering the test set size in the cross-validations is just 1/5 of the size of total population, we combined the environment-specific genomic predicted genetic values of the lines in the five test sets per repeat of cross-validation and used the Pearson correlation coefficient between the combined genomic predicted genetic values and corresponding environment-specific BLUEs to assess the genomic prediction accuracy. To statistically compare the prediction accuracies of different genomic prediction models, we firstly transformed the accuracies (correlations) using Fisher's z transformation i.e., z = 0.5 × ln((1 + r)/(1 − r)) where r is the correlation. The Student’s t-test was used to test the difference based on the transformed correlations.

## Result

### Phenotypic Data Quality and Genetic Diversity

The repeatability estimates of DTH in each environment of both the first and the second populations were all higher than 0.9 (Additional file 1: Table S1). The repeatability estimates of PH in each environment ranged from 0.744 to 0.886 in the first population, and were all above 0.8 in the second population (Additional file 1: Table S1). The heritability estimate of DTH was lowest (0.762) in the third population and highest (0.915) in the first population (Additional file 1: Table S1). For PH, the heritability estimates were similar in all populations, which were around 0.9 (Additional file 1: Table S1). For both DTH and PH, the distributions of environment-specific BLUEs of genetic effects of lines were asymptotically normal for most environments in the three populations (Additional file 2: Figs. S1–S3). The first and third populations were more diverse in contrast to the second population (Additional file 2: Fig. S4). No conspicuous families or subpopulations was observed in all the three populations (Additional file 2: Fig. S4).

### Haplotype Blocks Identified

**Fig. 2:**
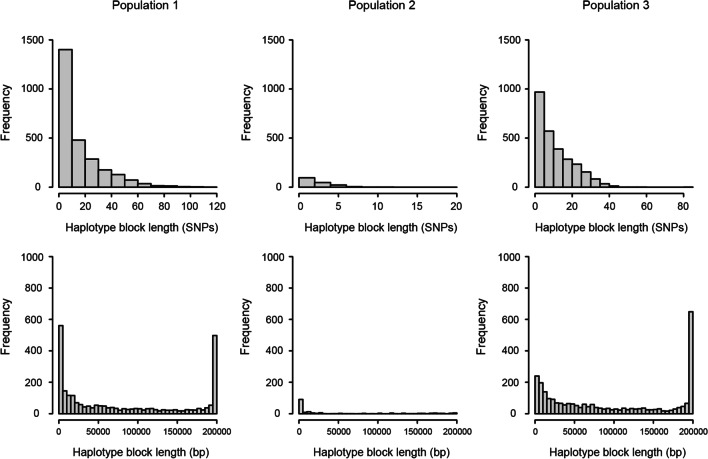
The frequency distribution of haplotype block length measured by the number of SNPs included (SNPs) and base pairs (bp) in the three populations

The number of haplotype blocks identified in the first to third population were 2620, 176, and 2740 respectively. The proportion of SNPs included in the haplotype blocks was 96.3%, 47.1%, and 96.3% respectively for the first to third population (Table 1). The shortest haplotype block of each of the three populations invariably consisted of 2 SNPs. The longest haplotype block identified in the first population included 116 SNPs while a large proportion of haplotype blocks contained no more than 10 SNPs. For the second and third population, the largest haplotype block respectively contained 12 and 83 SNPs (Fig. 2). There were a large number of long haplotype blocks of more than 195,000 base pair (bp) identified in the first and third populations. The number of short haplotype blocks of less than 5000 bp found in the first population was much more than that identified in the third population (Fig. 2).

**Table 1:** The size and genomic data statistic of each rice population

Population	The number of lines	The number of SNPs	The number of haplotype blocks identified	Proportion of SNPs in haplotype blocks (%)
Population 1	344	44,116	2620	96.3
Population 2	254	1193	176	47.1
Population 3	1048	33,518	2740	96.3

### Genomic Prediction Accuracies of Genotyped- and Haplotype-Based Models with Training Set of Different Phenotyping Intensities

**Fig. 3:**
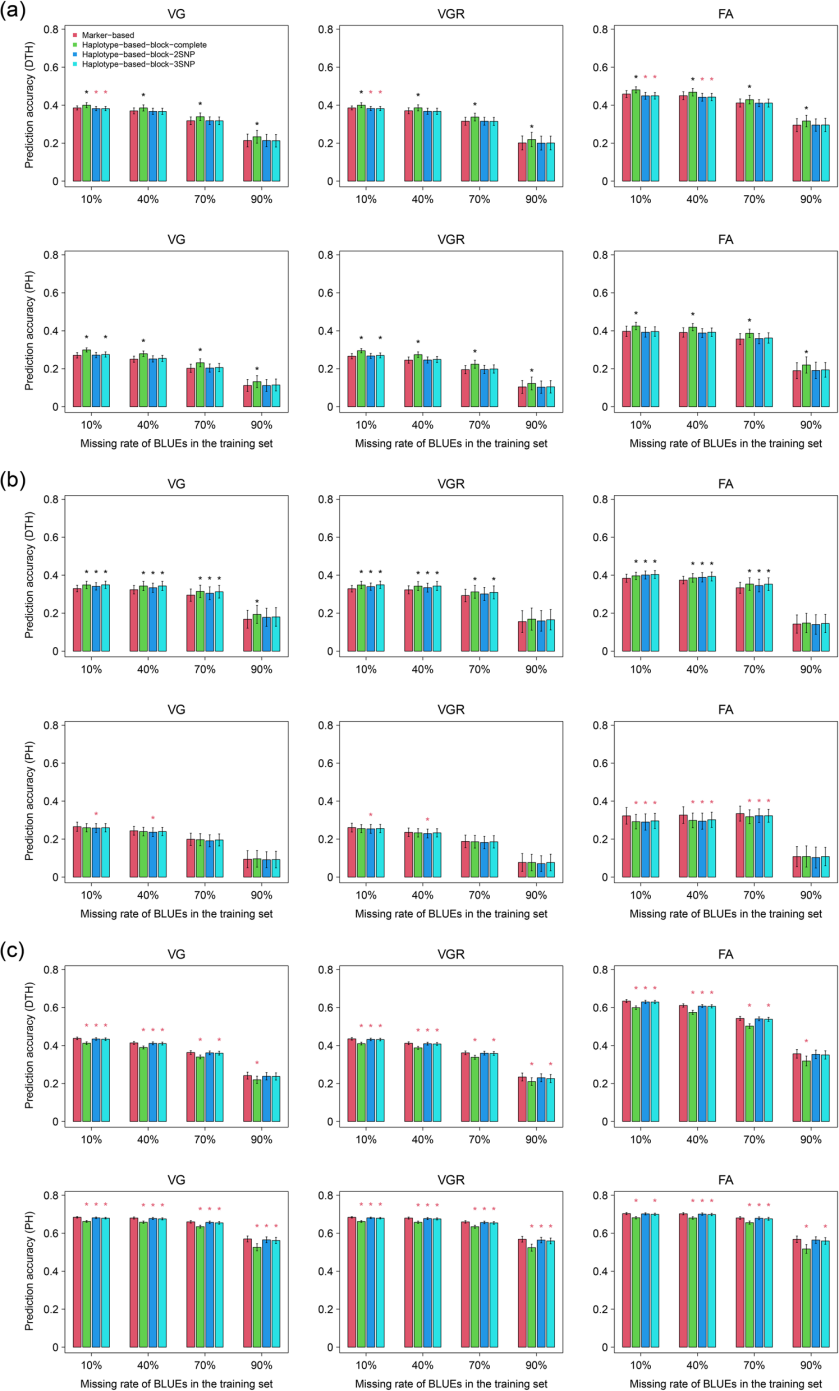
Genomic prediction accuracies of days to heading (DTH) and plant height (PH) in the **a** first, **b** second, and **c** third populations using three prediction models (VG, VGR, FA) with different missing rates of environment-specific best linear unbiased estimates (BLUEs) of genetic effects of lines in the training set. The genetic effect was respectively described by SNP genotypes (Marker-based), haplotype using complete blocks (Haplotype-based-block-complete), and short haplotypes containing two SNPs (Haplotype-based-block-2SNP) and three SNPs (Haplotype-based-block-3SNP) within a haplotype block. The whiskers at the top of the bars indicate the standard deviations of prediction accuracies in different cross-validation repeats. The asterisks above the bars indicate the prediction accuracies of haplotype-based approaches were statistically significantly (*p* < 0.05, t-test) higher (black) and lower (red) than those of the marker-based approach after a Fisher's z transformation

The genomic prediction accuracies were overall declining as the missing rates of environment-specific BLUEs increased irrelevant to the population, prediction model, and trait (Fig. 3; Additional file 3: Table S2). For both the traits studied, there was mostly no distinct decrease of prediction accuracy until the missing rate of training set rose to 70% disregarding the population and prediction approach (Fig. 3; Additional file 3: Table S2).

Comparing the predictive ability of different genomic prediction models, in the first population, for both DTH and PH, the prediction accuracies of FA model which is able to accommodate the covariances between environments were apparently higher than those of VG and VGR both considering no environmental correlation. The advantage of FA model over VG and VGR approaches was more conspicuous in PH than that in DTH. VG and VGR performed similarly indicating an inconsequential genotype-by-environment interaction effect (Fig. 3a; Additional file 3: Table S2). In the second population, FA model also outperformed VG and VGR approaches in both DTH and PH while not as distinct as that in the first population. The genotype-by-environment interaction was also inconspicuous inspecting the prediction accuracies of VG and VGR approaches. The decrease of prediction accuracy for training set including more than 70% of missing data in FA model for both traits was more evident as compared to those observed in VG and VGR approaches (Fig. 3b; Additional file 3: Table S2). In the third population, thanks to the large population size, prediction accuracies of all models were overall improved for both PH and DTH. The superiority of FA model over VG and VGR approaches was still present in DTH but disappeared in PH (Fig. 3c; Additional file 3: Table S2). The variation of prediction accuracies for all models in both traits was generally in line with the size of each population (Fig. 3; Additional file 3: Table S2).

For the different ways describing the genetic effect, in the first population, the prediction accuracies of haplotype-based approaches using complete haplotype blocks were all significantly (*p* < 0.05, t-test) higher than those of marker-based model disregarding the trait and phenotyping intensity of training set. There was no benefit of segmenting the haplotype blocks into small fragments with two or three SNPs despite the VG and VGR models using small haplotype blocks occasionally outperformed their marker-based counterparts in PH (Fig. 3a; Additional file 3: Table S2). In the second population, the haplotype-based approaches irrespective of using complete or segmented blocks were universally significantly (*p* < 0.05, t-test) superior to the marker-based models in DTH except for a high phenotypic missing rate in the training set. As compared, the image in PH was conversed that the haplotype-based methods were basically inferior to the marker-based model especially for the FA model (Fig. 3b; Additional file 3: Table S2). In the third population, the prediction accuracies of haplotype-based methods were comprehensively lower than their marker-based counterparts irrelevant to the trait and phenotyping intensity of training set. However, using small haplotype segments with two or three SNPs was able to greatly compensate the loss by using haplotypes instead of marker genotypes in the models, which almost caught up with marker-based models though a statistically significant difference of prediction accuracies was shown (Fig. 3c; Additional file 3: Table S2).

## Discussion

### Phenotyping Intensity of 30% is Sufficient for a Sparse Phenotyping Multi-Environment Training Set

The genomic prediction enhanced sparse phenotyping holds a huge potential in plant breeding in terms of reducing overall phenotyping cost of MET or multi-trait and increasing the number of environments for phenotyping or traits of interest without additional expense (He et al. 2021; Jarquín et al. 2020). The ways to utilize the sparse phenotyping were 1) from the calibration set selecting a subset of reference genotypes that are most representative of total calibration set or most genetically close to the individuals in the test set to form the training set (Akdemir et al. 2015; Isidro et al. 2015; Rincent et al. 2012); 2) mimicking the breeding situations that a subset of individuals are phenotyped in MET to predict the performance of untested genotypes (CV1), or the individuals are evaluated in some environments or for some traits and the missing records in the MET or multi-trait dataset are predicted based on the observations (CV2) (Burgueño et al. 2012; Jarquín et al. 2014). The cross-validation scheme with sparse phenotyping of training set could be regarded as an extension of CV1 in which the training set is in addition parsimoniously phenotyped. It was observed that maintaining merely 30% phenotypic records in the multi-environment training set was able to achieve a comparable prediction accuracy to high phenotyping intensities for both DTH and PH in all the three populations used in our study (Fig. 3; Additional file 3: Table S2). As we performed a random masking for the phenotypic records in the multi-environment training set, the optimal trade-off phenotyping intensity, i.e., 30%, could be further reduced by composing the training set using the most related genotypes to a specific test set (Akdemir et al. 2015; Isidro et al. 2015; Rincent et al. 2012) and optimizing the resource allocation in the sparse phenotyping to fully exploit the environmental correlations (Atanda et al. 2022; Jarquín et al. 2020). It is also worth to investigate the relevance of the training set size to the optimal sparse phenotyping intensity, relative to the 5-fold cross-validation scheme adopted in our study with a fixed number of reference and test lines.

### Segmenting the Haplotype Blocks into Small Fragments for Genomic Prediction is Recommended Especially in a Large Population

Theoretically, the haplotype-based models should outperform their marker-based counterparts as the local epistatic effects are accommodated in addition to the additive effects (Jiang et al. 2018). However, using a rice public dataset including 413 varieties phenotyped for 26 traits the authors showed that the haplotype-based approach using small haplotypes with varying lengths of two to ten SNPs was generally advantageous for DTH, but not for PH (Jiang et al. 2018). This result is in accordance to our findings in the second population (Fig. 3b; Additional file 3: Table S2). Actually, only the performances of marker-based and haplotype-based models in the second population were comparable as the number of available molecular variants in different models are alike (Tables 1, 2).

**Table 2:** The number of molecular variants available in different haplotype-based approaches in each rice population

Population	Haplotype-based-block-complete	Haplotype-based-block-2SNP	Haplotype-based-block-3SNP
Population 1	9054	51,482	38,494
Population 2	969	1183	1082
Population 3	10,186	39,230	29,882

Haplotype-based-block-complete: haplotype-based genomic prediction model using complete haplotype blocks; Haplotype-based-block-2SNP: haplotype-based genomic prediction model using small haplotype fragments composed by two SNPs; Haplotype-based-block-3SNP: haplotype-based genomic prediction model using small haplotype fragments composed by three SNPs

It was shown that the haplotype-based model using complete haplotype blocks was superior for both DTH and PH in the first population consisting of a few hundred genotypes despite the lower number of available variants in the haplotype-based model using complete blocks relative to those in the marker-based model and haplotype-based model with segmented haplotypes (Fig. 3a; Tables 1, 2; Additional file 3: Table S2). The phenomenon that using more molecular variants would have a negative impact on the prediction accuracy was also observed by the data providers in their study (Spindel et al. 2015). It means, for a small population, a large number of molecular variants would be unnecessary. By comparison, the third population is large comprising more than one thousand lines, in which abundant SNPs are favoured. Thus, the deficiency of number of available molecular variants in the prediction models would be the reason for the conspicuously low prediction accuracy of haplotype-based model using complete block relative to the marker-based model. In this case, segmenting the complete haplotype blocks into small fragments which could keep a sufficient number of haplotypes available holds the potential to improve the prediction accuracy through capturing the local epistatic effects compared to the marker-based model (Fig. 3c; Tables 1, 2; Additional file 3: Table S2). However, in our study, no benefit was observed by using the haplotype-based models with segmented haplotype blocks. In the second population, the superiority of haplotype-based models over the marker-based model was exclusively observed for DTH (Fig. 3b; Additional file 3: Table S2). This might be due to the local epistatic effects prevailing in long haplotypes while the number of available haplotypes from complete haplotype blocks is insufficient, thereby the advantage of modelling local epistatic effects could not be reflected on the prediction accuracy. This hypothesis could be corroborated by Jiang et al. (2018) in which the highest prediction accuracy of haplotype-based model for DTH was achieved when seven SNPs were used to compose the haplotypes.

More studies using larger rice populations and other critical traits such as grain yield are necessitated to validate the potential of haplotype-based genomic prediction using complete and segmented haplotype blocks.

### Modelling Environmental Covariances in Genomic Prediction Improves Predictive Ability

It was observed that the genomic prediction model including genotype-by-environment interactions without the accommodation of environmental covariances, i.e., VGR, could not improve the prediction accuracy for both DTH and PH compared to the baseline model considering no genotype-by-environment interaction, i.e., VG (Fig. 3; Additional file 3: Table S2), which is in line with the findings in Ben Hassen et al. (2018) and Cui et al. (2020). It could be accounted for by the fact that the cross-validation scheme, i.e., CV1, we adopted is not as robust as CV2 with a dispersed phenotype missing pattern in modelling genotype-by-environment interaction because Ben Hassen et al. (2018) observed a conspicuous increase of prediction accuracy when CV2 was examined in place of CV1. A similar result was shown in Monteverde et al. (2019) that the reaction norm models, like the approaches used in our study, including genotype-by-environment interactions was not superior to the models without interactions in a “leave one environment out” scenario aiming to predict the performance of genotypes in new environments.

Modelling the covariances between environments in multi-environment genomic prediction was overall advantageous in our study (Fig. 3; Additional file 3: Table S2). The extent of the benefit depends on the trait and population. Referring to the literature, Monteverde et al. (2018) found using an unstructured environmental covariance matrix could slightly improve the prediction accuracy of PH in an *indica* population under scenario CV1 in contrast to a diagonal variance–covariance structure assuming no correlations between environments. Unsurprisingly, the extent of improvement on prediction accuracy by modelling correlations between environments was remarkably boosted in scenario CV2. Ben Hassen et al. (2018) compared the predictive abilities of Reproducing Kernel Hilbert Space (RKHS) approaches with and without the consideration of genetic correlations between environments and found no noticeable difference between them for three studied traits including DTH. Jarquín et al. (2014) revealed a great potential of using environmental covariates (enviromics data) to specifically portray the environments in a reaction norm model in wheat. The potential was validated by Monteverde et al. (2019) in a *japonica* rice population using reaction norm models. In the *indica* population, another genomic prediction approach namely partial least square regression (PLS) held the superiority of using environmental covariates to depict environments and their relationships.

Wang et al. (2017) proposed to utilize the multi-environment phenotypic values as phenomics data in genomic prediction models to account for the phenotypic variance not explained by the genetic effect. It was shown that within a target environment the prediction of rice hybrids in the test set was marginally enhanced by fitting a phenomics kernel in the single environment model. Taking the advantage of using enviromics data to describe the environments together, the modern plant breeding technology should be an integrated approach efficiently making use of multi-omics information (Crossa et al. 2021).

## Conclusion

The multi-environment genomic prediction can help to discover the elite rice varieties resilient to diverse environments or particularly suited to a specific environment. To improve the efficiency of multi-environment genomic prediction, sparse phenotyping can be used to establish a multi-environment training set. We demonstrated that a 30% phenotyping intensity in the multi-environment training set is sufficient to provide a comparable prediction accuracy to high phenotyping intensities for traits like PH and DTH. Basing on LD to identify haplotype blocks and accordingly making haplotypes for genomic prediction could capture local epistatic effects more reasonably compared to a fixed length of haplotypes. We demonstrated that the haplotype-based models are worth to be implemented in the prediction of DTH and segmenting the haplotype blocks into small fragments with two or three SNPs could maintain the predictive ability of haplotype-based models in large populations. Modelling the covariances between environments improves multi-environment genomic prediction accuracy irrespective of capitalizing on marker genotypes or haplotypes.

## Supplementary Information

**Additional file 1**: **Table S1**. Heritabilitiesand repeatabilities in each environment for days to headingand plant heightin the three rice populations.

**Additional file 2**: **Fig. S1**. Distribution of best linear unbased estimatesof genetic values of lines fordays to headingandplant heightin each environmentof the first population. The title of each barplot indicates the environment. **Fig. S2**. Distribution of best linear unbased estimatesof genetic values of lines fordays to headingandplant heightin each environmentof the second population. The title of each barplot indicates the environment. **Fig. S3**. Distribution of best linear unbased estimatesof genetic values of lines fordays to headingandplant heightin each environmentof the third population. The title of each barplot indicates the environment. **Fig. S4**. Genetic diversity of thefirst,second, andthird populations described by Euclidean distance between lines based on SNP genotypic scores. The average clustering method was used to order the lines.

**Additional file 3**: **Table S2**. Genomic prediction accuraciesof days to headingand plant heightin the three rice populations using three prediction modelswith different missing rates of environment-specific best linear unbiased estimatesof genetic effects of lines in the training set. The genetic effect was respectively described by SNP genotypes, haplotype using complete blocks, and short haplotypes containing two SNPsand three SNPswithin a haplotype block. The missing rate zeroindicates the training set holds complete environment-specific BLUEs. The asterisk and pound sign indicate the prediction accuracies of haplotype-based approaches were statistically significantlyhigher and lower than those of the marker-based approach.

## Data Availability

The data of first population is publicly available in the Data Dryad digital repository, datadryad.org/stash/dataset/doi:105061/dryad.7369p. The request for the data of second and third populations should be directed to the corresponding author.

## Abbreviations

MET: Multi-environment trials
LD: Linkage disequilibrium
DTH: Days to heading
PH: Plant height
SNPs: Single nucleotide polymorphisms
GEBVs: Genomic estimated breeding values
DS: Dry season
WS: Wet season
RILs: Recombinant inbred lines
IRRI: International rice research institute
JX: Jiangxi
SZ: Shenzhen
BLUEs: Best linear unbiased estimates
BLUP: Best linear unbiased predictions
FA: Factorial analytic
CV: Cross-validation
