# Supplementary material for: Sparse Phenotyping and Haplotype-Based Models for Genomic Prediction in Rice
Source: Rice (N Y). 2023 Jun 7;16:27. doi: 10.1186/s12284-023-00643-2 (PMC10247604; doi:10.1186/s12284-023-00643-2)
Supplement: Supplementary file 1 — Additional file 1: Table S1. Heritabilitiesand repeatabilities in each environment for days to headingand plant heightin the three rice populations. [file 12284_2023_643_MOESM1_ESM.docx]

**Table S1** Heritabilities (h^2^) and repeatabilities in each environment for days to heading (DTH) and plant height (PH) in the three rice populations

| Population | Environment | DTH | PH |
| --- | --- | --- | --- |
| Population 1 | 2009DS | 0.955 | 0.802 |
|  | 2009WS | 0.949 |  |
|  | 2010DS | 0.97 | 0.886 |
|  | 2010WS | 0.934 | 0.758 |
|  | 2011DS | 0.958 | 0.862 |
|  | 2011WS | 0.934 | 0.753 |
|  | 2012DS | 0.941 | 0.744 |
|  | 2012WS | 0.944 | 0.875 |
|  | h^2^ | 0.915 | 0.909 |
| Population 2 | DS1 | 0.926 | 0.884 |
|  | DS2 | 0.91 | 0.865 |
|  | WS1 | 0.929 | 0.875 |
|  | WS3 | 0.917 | 0.887 |
|  | h^2^ | 0.867 | 0.915 |
| Population 3 | h^2^ | 0.762 | 0.895 |
