# Supplementary material for: Sparse Phenotyping and Haplotype-Based Models for Genomic Prediction in Rice
Source: Rice (N Y). 2023 Jun 7;16:27. doi: 10.1186/s12284-023-00643-2 (PMC10247604; doi:10.1186/s12284-023-00643-2)
Supplement: Supplementary file 2 — Additional file 2: Fig. S1. Distribution of best linear unbased estimatesof genetic values of lines fordays to headingandplant heightin each environmentof the first population. The title of each barplot indicates the environment. Fig. S2. Distribution of best linear unbased estimatesof genetic values of lines fordays to headingandplant heightin each environmentof the second population. The title of each barplot indicates the environment. Fig. S3. Distribution of best linear unbased estimatesof genetic values of lines fordays to headingandplant heightin each environmentof the third population. The title of each barplot indicates the environment. Fig. S4. Genetic diversity of thefirst,second, andthird populations described by Euclidean distance between lines based on SNP genotypic scores. The average clustering method was used to order the lines. [file 12284_2023_643_MOESM2_ESM.docx]

**
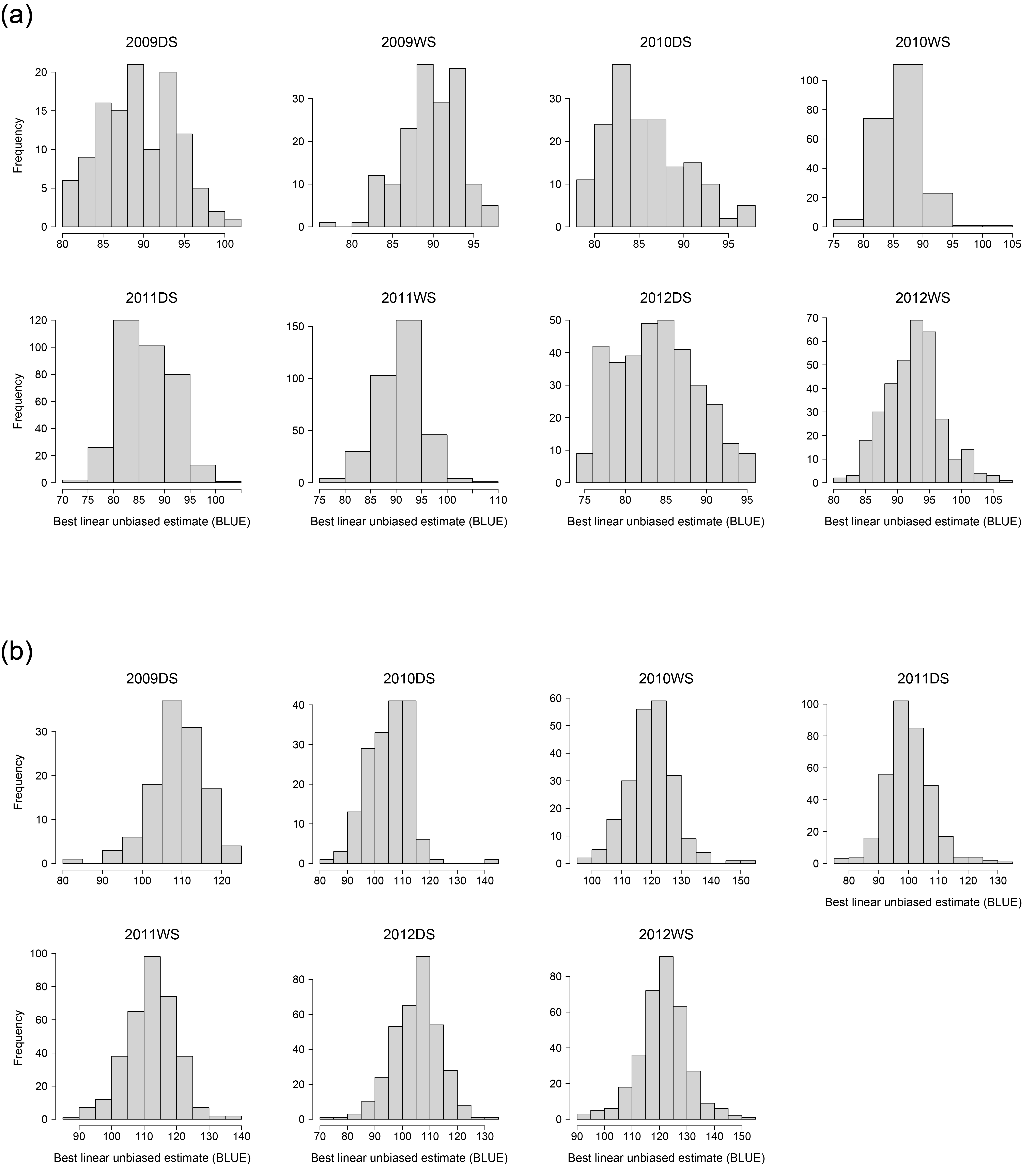
Fig. S1** Distribution of best linear unbased estimates (BLUEs) of genetic values of lines for (a) days to heading (DTH) and (b) plant height (PH) in each environment (year-season combination) of the first population. The title of each barplot indicates the environment

**
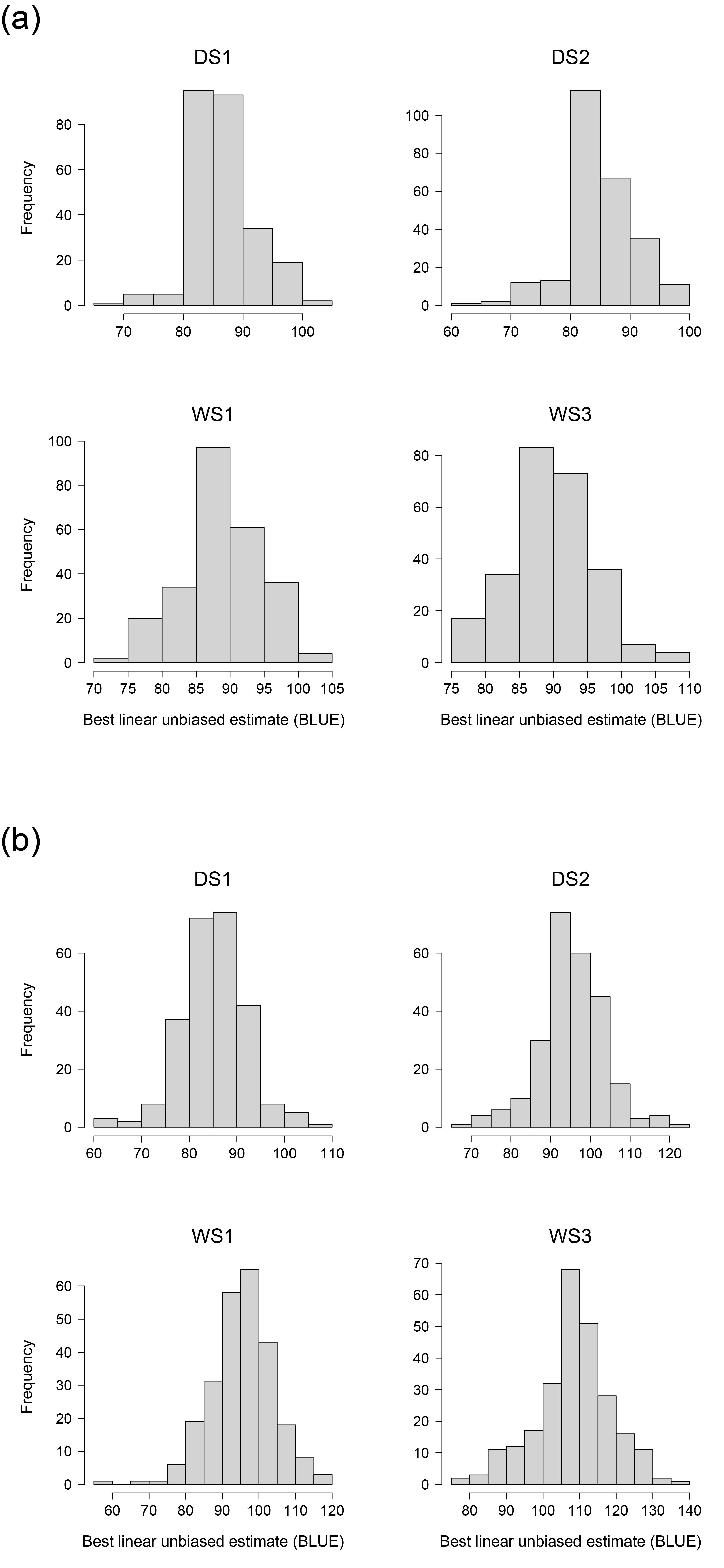
Fig. S2** Distribution of best linear unbased estimates (BLUEs) of genetic values of lines for (a) days to heading (DTH) and (b) plant height (PH) in each environment (the combination of season and nitrogen fertilizer application rate) of the second population. The title of each barplot indicates the environment

**
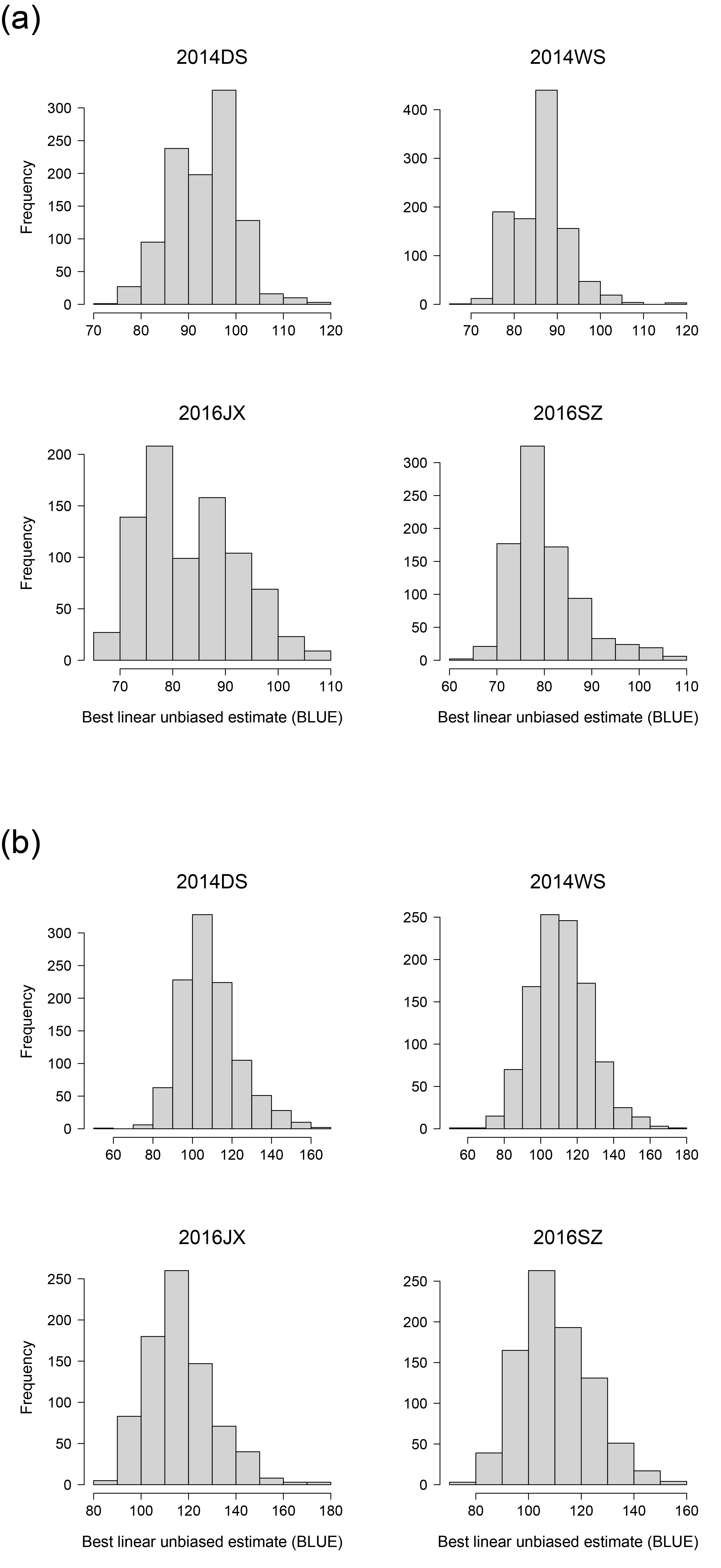
Fig. S3** Distribution of best linear unbased estimates (BLUEs) of genetic values of lines for (a) days to heading (DTH) and (b) plant height (PH) in each environment (the combination of season/location and year) of the third population. The title of each barplot indicates the environment

**
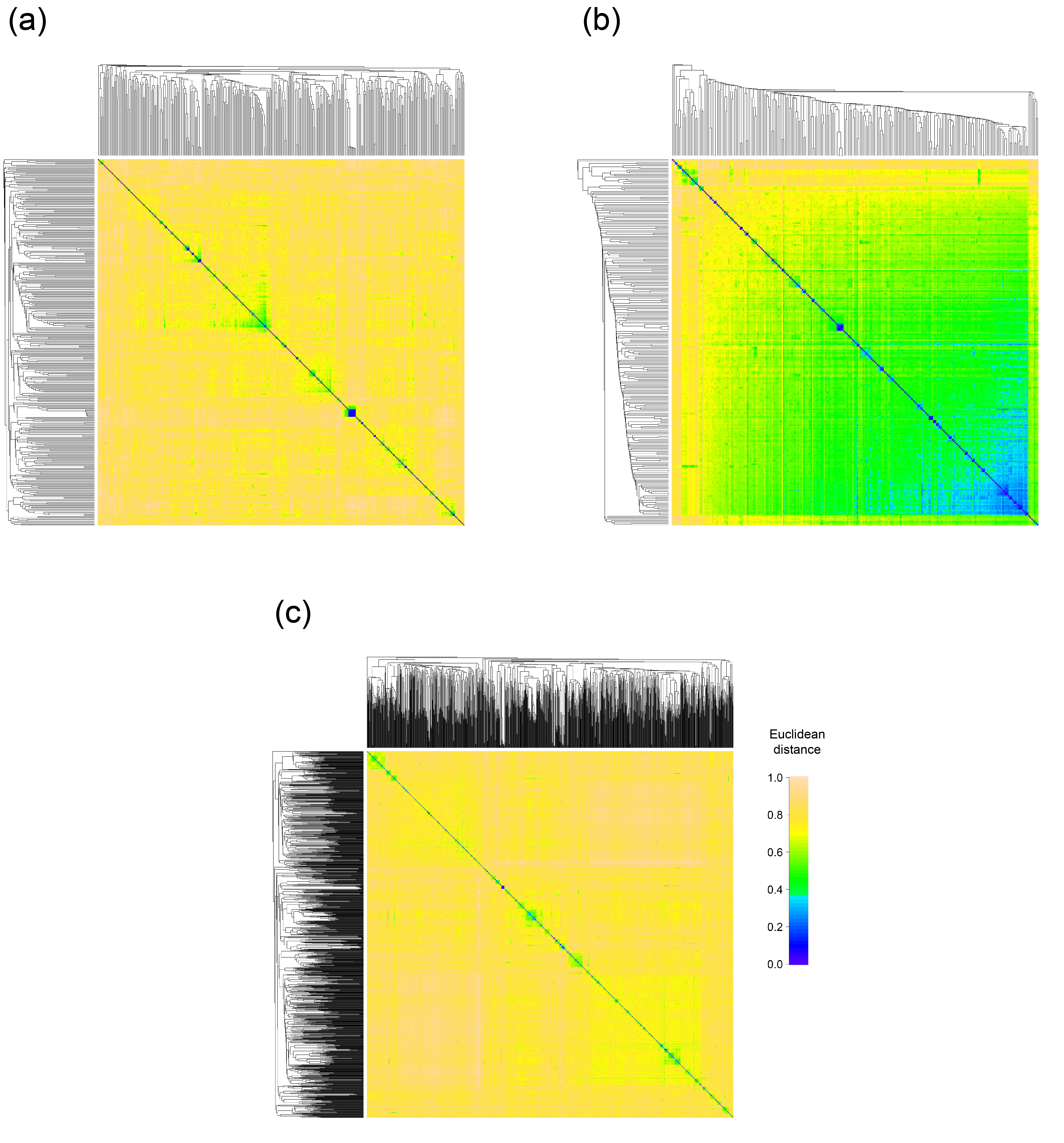
Fig. S4** Genetic diversity of the (a) first, (b) second, and (c) third populations described by Euclidean distance between lines based on SNP genotypic scores. The average clustering method was used to order the lines
